# Inhibition of (Pro)renin Receptor-Mediated Oxidative Stress Alleviates Doxorubicin-Induced Heart Failure

**DOI:** 10.3389/fonc.2022.874852

**Published:** 2022-04-29

**Authors:** Xiao-yi Du, Dao-chun Xiang, Ping Gao, Hua Peng, Ya-li Liu

**Affiliations:** ^1^ Department of Pediatrics, Union Hospital, Tongji Medical College, Huazhong University of Science and Technology, Wuhan, China; ^2^ Department of Pediatrics, Maternal and Child Hospital of Hubei Province, Tongji Medical College, Huazhong University of Science and Technology, Wuhan, China; ^3^ Department of Pharmacy, The Central Hospital of Wuhan, Tongji Medical College, Huazhong University of Science and Technology, Wuhan, China; ^4^ Department of Clinical Pharmacy, Wuhan Children’s Hospital, Tongji Medical College, Huazhong University of Science and Technology, Wuhan, China

**Keywords:** doxorubicin, cardiotoxicity, heart failure, (Pro) renin receptor, RAC1

## Abstract

**Aim:**

Clinical utility of doxorubicin (DOX) is limited by its cardiotoxic side effect, and the underlying mechanism still needs to be fully elucidated. This research aimed to examine the role of (pro)renin receptor (PRR) in DOX-induced heart failure (HF) and its underlying mechanism.

**Main Methods:**

Sprague Dawley (SD) rats were injected with an accumulative dosage of DOX (15 mg/kg) to induce HF. Cardiac functions were detected by transthoracic echocardiography examination. The levels of lactate dehydrogenase (LDH) and creatine kinase (CK) in serum were detected, and oxidative stress related injuries were evaluated. Furthermore, the mRNA expression of *PRR* gene and its related genes were detected by real-time PCR (RT-PCR), and protein levels of PRR, RAC1, NOX4 and NOX2 were determined by Western blot. Reactive oxygen species (ROS) were determined in DOX-treated rats or cells. Additionally, PRR and RAC1 were silenced with their respective siRNAs to validate the *in vitro* impacts of PRR/RAC1 on DOX-induced cardiotoxicity. Moreover, inhibitors of PRR and RAC1 were used to validate their effects *in vivo*.

**Key Findings:**

*PRR* and *RAC1* expressions increased in DOX-induced HF. The levels of CK and LDH as well as oxidative stress indicators increased significantly after DOX treatment. Oxidative injury and apoptosis of cardiomyocytes were attenuated both *in vivo* and *in vitro* upon suppression of PRR or RAC1. Furthermore, the inhibition of PRR could significantly down-regulate the expressions of RAC1 and NOX4 but not that of NOX2, while the inhibition of RAC1 did not affect PRR.

**Significance:**

Our findings showed that PRR inhibition could weaken RAC1-NOX4 pathway and alleviate DOX-induced HF *via* decreasing ROS production, thereby suggesting a promising target for the treatment of DOX-induced HF.

**Graphical Abstract:**
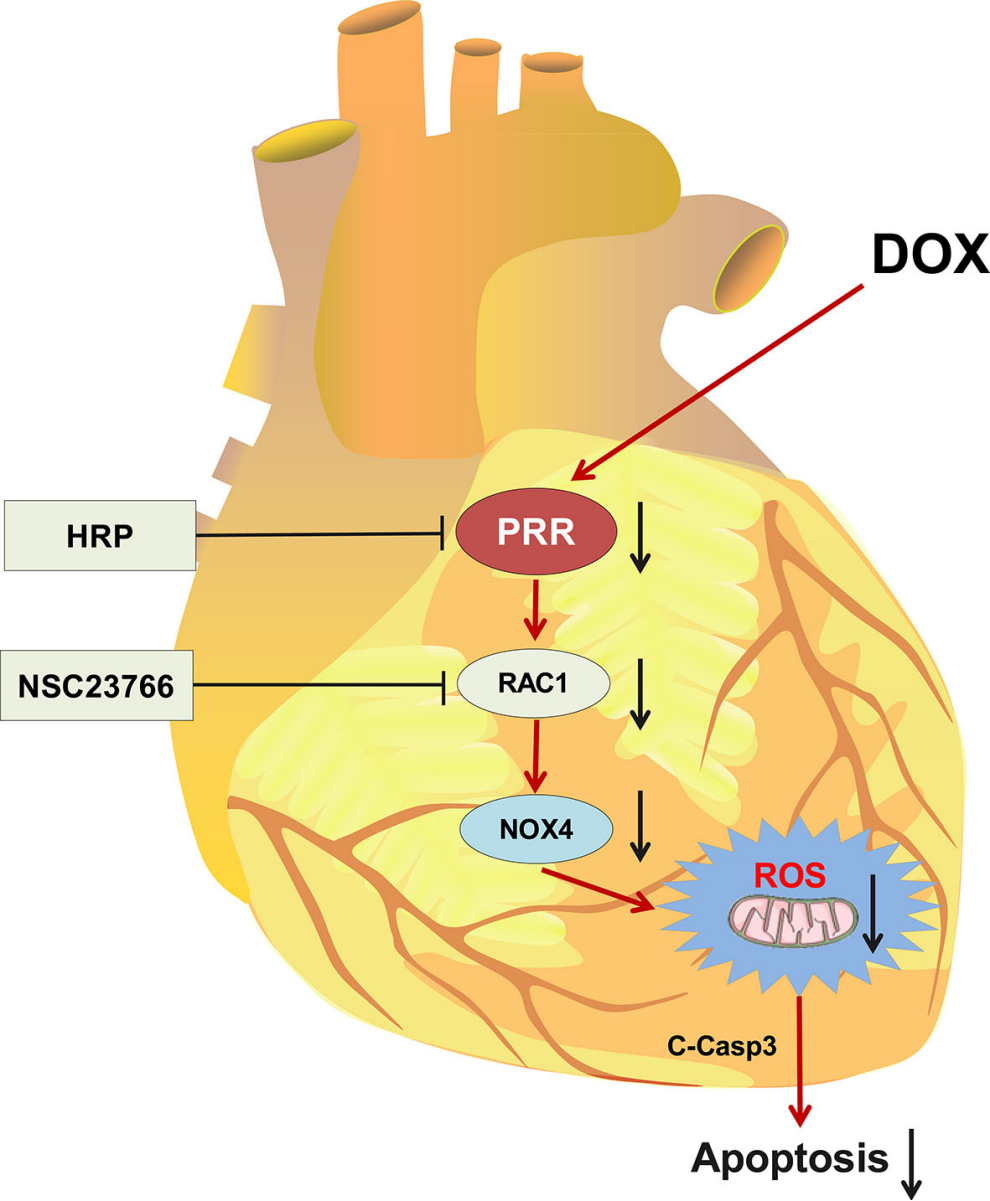


## Introduction

Doxorubicin (DOX) is a classical anthracyclines chemotherapeutic drug with wide clinical utility for acute leukemia, breast cancer, lymphoma, and ovarian cancer (1, 2). However, most patients receiving DOX chemotherapy are prone to develop acute, subacute, early or late cardiotoxicity (3, 4). DOX-induced serious cardiovascular complications mainly include hypotension, tachycardia, arrhythmia, ventricular dysfunction, and heart failure (HF) (4–7). Among them, HF is the most serious side effect which lead to a 3.5-fold increased mortality risk comparing to another idiopathic cardiomyopathy. The HF caused by DOX is dose-dependent, and its incidence increased to 48% as the lifetime accumulation dose of DOX increases to 700 mg/m^2^ (8, 9). This toxicology may lead to premature morbidity and even death among those cancer survivors (9). But the treatment to DOX-induced HF is still limited at present and the mechanism still need to be fully elucidated.

Several studies suggest that DOX-induced myocardial damage involved in numerous alterations, such as mitochondrial injury, DNA damage, lipid peroxidation, mitochondrial injury, autophagy, oxidative stress, and apoptosis (2, 10). It is widely accepted that oxidative stress is a critical process in the development of DOX-induced HF (2, 11). DOX intake produces massive amounts of reactive oxygen species (ROS), thereby resulting in the impairment of mitochondrial function and damage to cells (12, 13). As a consequence, inhibiting oxidative stress might serve as a promising preventive measure against DOX-induced HF; however, the underlying mechanism of DOX-triggered oxidative injury remains unknown.

The (pro)renin receptor (PRR), also called APT6AP2, is a crucial modulators of ROS production (14, 15). By binding to pro-renin and renin, PRR has been shown to activate the rennin-angiotensin pathway and the binding process triggers the receptor itself, which is involved in a variety of intracellular physiological processes (11, 14, 16). Recently, PRR is thought to play a crucial role in heart diseases. Mahmud et al. reported overexpression of PRR in myocardial infarction animal models or the biopsy samples obtained from patients with dilated cardiomyopathy (17). And a previous study showed that overexpression of PRR in mice heart leads to atrial fibrillation (18). Besides, PRR-activated Ras-related C3 botulinum toxin substrate 1(RAC1) in smooth muscle cells may participate in the pathogenesis of various diseases (19), and the activation of Rac1 contributes to oxidative stress-related injury in the myocardium (20). However, it is unclear whether PRR play a role in the DOX-induced cardiotoxicity. Therefore, the purpose of the present study was to examine the effects of PRR in DOX-induced HF and investigate its underlying mechanism.

## Materials and Methods

### Reagents

An antibody directed against PRR (#A6531) was purchased from ABclonal (Cambridge, MA, USA). Anti-Rac1 antibody (#AF4200) and NADPH oxidase 4 (NOX4) (#DF6924) were obtained from Affinity Biosciences (OH, USA). Cleaved-caspase 3 (#9664) was procured from Cell Signaling Technology (MA, USA). Anti-GAPDH antibody (Abs132004a) was obtained from Absin BioscienceInc (Shanghai, China). An *in-situ* cell death detection kit (11684817910) (Roche, Penzberg, Germany) was used to perform the terminal deoxynucleotidyl transferase-mediated dUTP-biotin nick end labeling assay (TUNEL) assay. Rabbit polyclonal antibodies against CD31 were purchased from Abcam (Cambridge, UK). Dihydroethidium (DHE, D11347) was purchased from Molecular Probes Invitrogen (Carlsbad, CA, USA). 2,7-Dichlorodi-hydrofluorescein diacetate (DCFH-DA, S0033S) was purchased from Beyotime Institute of Biotechnology (Shanghai, China). The PRR inhibitor, handle region peptide (HRP), was obtained from Chinapeptides Co. Ltd. (Shanghai, China). The bicinchoninic acid (BCA) protein assay kit was obtained from Wuhan Kerui Technology Co. Ltd. (Wuhan, China). Cell counting kit 8 (CCK-8) was purchased from Dojindo Molecular Technologies, Inc. (MD, USA). The detection kits for lactate dehydrogenase (LDH, A020-1-1), creatine kinase (CK, A032-1-1), and oxidative stress-related enzymes, such as malondialdehyde (MDA, A003-1-2), superoxide dismutase (SOD, A001-3-2), glutathione (GSH, A006-2-1) and glutathione peroxidase (GSH-Px, A005-1-2) were purchased from the Nanjing Jiancheng Institute of Biotechnology (Nanjing, China).

### Animals and Experimental Protocols

Male Sprague Dawley (SD) rats (SPF Biotechnology Co., Ltd., Beijing, China) were reared in specific pathogen-free (SPF) environment at the Experimental Animal Center of Tongji Medical College, Huazhong University of Science and Technology (Wuhan, China). Approval of all the experimental designs involving animals was granted by the Animal Ethics Committee of Tongji Medical College, Huazhong University of Science and Technology (Wuhan, China). After a week of adaptive feeding, animals in the DOX group were administered intraperitoneally with DOX at the dose of 2.5 mg/kg over a period of 2 weeks for an accumulative dosage of 15 mg/kg (21), whereas control rats were inoculated with an equivalent amount of 0.9% saline. Then eight days later, an osmotic minipump (2ML4, Alzet, CA, USA) was implanted subcutaneously for the infusion of the vehicle and HRP (0.1mg/kg/d for 7 successive days) under isoflurane anesthesia. NSC23766 (2.5 mg/kg/d) was administered *via* an intraperitoneal injection for the same duration (seven consecutive days) (n=8) (22). Finally, echocardiography was performed before the rats were sacrificed. Serum samples were collected, and heart tissues were immediately excised for further experiments.

### Echocardiography

Rats received isoflurane anesthesia and then fixed on a plat board. Next, trans-thoracic and M-mode echocardiographic studies were performed using the 25.0 MHz Intelligent color Doppler echocardiography (EPIQ 7C) platform with L12-3 probes (Philips, Eindhoven, the Netherlands) at the end of the posttreatment period. Left ventricular functions, including fractional shortening (FS), left ventricular end-diastolic diameter (LVEDD), left ventricular ejection fraction (LVEF), and left ventricular end-systolic diameter (LVESD) was computed as described previously (23). For all measurements, an average of 3 successive cardiac cycles was evaluated. The LVEF was calculated using the following equation: LVEF (%) = (LVEDV - LVESV)/LVEDV×100%.

### Histological Analysis

After sacrificing the rats, their left ventricles were collected and immediately fixed in 4% buffered paraformaldehyde. After embedding the heart tissue in paraffin and cutting it into 5 μm-thick slices, hematoxylin and eosin (H&E) staining was performed to examine the heart pathology. From each section, images of the heart tissues were captured using an optical microscope (Olympus, Tokyo, Japan; magnification 200×). Histopathological analysis was performed for randomly selected cortical fields and images were examined by a pathologist who was blinded to the treatment groups.

### Detection of CD31^+^ Cells

The immunohistochemical study was performed as Ammar, H. I. et al. described (24). After fixed by 10% formalin, cardiac samples were cut into 5 μm thick sections and air-dried overnight. After dewaxed in xylene, and rehydrated, the samples were for 15 minutes in antigen retrieval solution. Then the CD31 staining was performed by using primary antibody and secondary antibody. Then the sections were incubated with DAB at room temperature for 10 min. Finally, images were abstained for each sample using the microscope (Olympus, Japan) (magnification 400×).

### Cell Culture and Treatment

The rat cardiomyoblasts cell line, H9C2, was kindly donated by Dr. Yang Sun of the Tongji Hospital Affiliated with Tongji Medical College, Huazhong University of Science and Technology, (Wuhan, China). In a humid chamber containing 5 percent CO_2_ and 95 percent oxygen at a temperature of 37°C, the cells were maintained in Dulbecco’s Modified Eagle’s Medium (DMEM) (Hyclone, UT, USA) that contained 10 percent fetal bovine serum (FBS, Zhejiang Tianhang Biotechnology Co., Ltd., Hangzhou, China). H9C2 cells were subsequently plated at a density of 6 × 10^3^ cells/well and incubated for 24 hours. Next, DOX at varying concentrations (0-10 μM) was introduced to incubate the cells for 24h, and the optimal dose of DOX for subsequent analyses was identified.

### Cell Viability and Apoptosis Assays

CCK-8 assay kit was employed to measure the viability of the cells. The H9C2 cells were placed into 96-well plates at a density of 1×10^4^ cells/well and incubated for 24 hours. Following treatment, the medium from every well was replenished with 100 μL of DMEM comprising 10 percent CCK-8 reagent. Subsequently, the cells were subjected to incubation at 37°C for 2 hours. The absorbance of each sample was detected at 450 nm (Thermo Multiskan MK3 Microplate Reader, Thermo Fisher Scientific, MA, USA).

Cellular apoptosis assay was performed following a previously reported procedure (25). Briefly, after washed by PBS for three times, H9C2 cells were suspended and incubated in buffer with annexin V in the dark for 10 min. Following that, the H9C2 cells were incubated once again for 15 minutes using annexin V-FITC/propidium iodide. Finally, the H9C2 cells were analyzed utilizing flow cytometry (BD FACSCalibur, CA, USA) and the apoptosis was determined using FlowJo 10. All assays were performed in triplicates.

### Small Interfering RNA (siRNA) Transfection

H9C2 cells were seeded into 6-well plates for 24h, followed by incubation overnight at a temperature of 37°C and a CO_2_ concentration of 5%. Cells were transfected with siRNAs at 50-70% confluency. For silencing the PRR or RAC1 proteins, H9C2 cells were transfected with siRNAs constructs targeting PRR (RiboBio; Guangzhou, China) or RAC1 (Sigma-Aldrich, St Louis, USA.) for 24h before harvest according to the guidelines provided by the manufacturer. Scrambled siRNA (RiboBio; Guangzhou, China) was used as the control, and the sequence of negative control primers used in this study was as follows: 5’→3’: UUCUCCGAACGUGUCACGUTT (100 nM final); 3’→5’: ACGUGACACGUUCGGAGAATT (100 nM final). Furthermore, the transfection efficiency was analyzed as a measure of the knockdown.

### Real-Time PCR

Quantitative real-time reverse transcription polymerase chain reaction (RT–PCR) was conducted to verify the changes in mRNA expressions. Extraction of the total RNA from cardiac tissues was performed utilizing the TRIzol (Invitrogen, MO, USA) reagent. Total RNA (1 mg) from each sample was denatured at 65°C for 10 min. Subsequently, the cDNA was synthesized at 37°C for 1 h using a cDNA synthesis kit (GeneCopoeia, Rockville, MD, USA). The 2-△△CT technique was applied to assess relative levels of gene expressions. Below is a list of the primer sequences used in the present study: rat PRR (forward 5′-TCTGTTCTCAACTCGCTCC C-3 and reverse 5′-TCTCCATAACGCTTCCCAAG-3′); RAC1 (forward: 5′- CCTGCTCATCAGTTACACGACCA-3′, reverse: 5′-GTCCCAGAGGCCCAGATTCA-3′), Wnt3A (forward: 5′-ACCATGTTCGGGACCTATTCCA-3′, reverse: 5′-GCCTGTAGCATCTCGCTTCCA-3′); Wnt8A (forward: 5′-GGAGGCCAGGAGAGATG-3, reverse: 5′-ACGGAGACCACAAAAGGA-3′), NOX2 (forward: CTGCCAGTGTGTCGGAATCT

-3′, reverse: 5′-TGTGAATGGCCGTGTGAAGT-3′), NOX4 (forward: 5′- ATGTTGGGCCTAGGATTGTGT -3′, reverse: 5′- TCCTGCTAGGGACCTTCTGT -3′) and GAPDH (forward 5′-AAGTTCAACGGCACAGTCAA-3′ and reverse 5′- TCTCGCTCCTGGAAGATGG -3.)

### Western Blotting Analysis

The total proteins from the cardiac tissues were homogenized in RIPA lysis solution that contained the phosphatase inhibitors and protease. The protein content was detected by performing BCA protein quantitation assay. Next, SDS-PAGE was used to isolate the protein samples (40 µg/lane), which were then loaded onto a PVDF membrane. Following the blocking of the membrane using 5% non-fatty milk for 1h, it was subjected to incubation with the corresponding primary antibodies overnight at 4°C, as follows: GAPDH (1:1000), PRR (1:1,000), RAC1 (1:1,000), NOX2 (1:1,000), NOX4 (1:1,000), Cleaved-caspase 3 (1:1000), and thioredoxin 2 (1:1000). After incubation with secondary antibodies for 1h, the proteins were visualized on an ECL detection system (Syngene, UK). GAPDH was utilized to normalize the band intensity values.

### Detection of Serum Biochemical Indexes

In order to measure CK and LDH levels in serum, commercial kits were utilized in accordance with the manufacturer’s specifications. Additionally, the GSH-Px, MDA, GSH, and SOD levels in heart tissues were evaluated in accordance with the protocols described by the manufacturers.

### Measurement of Oxidant Species by DHE in Tissues or DCFH-DA in Cells

Oxidative stress levels in myocardial tissues, both *in vivo* and *in vitro* were measured as described previously (26). ROS production was determined by DHE or DCFH-DA staining. Briefly, after washing with phosphate-buffered saline (PBS), fresh myocardial tissues were frozen. Subsequently, the myocardial tissues were dissected into sections (5 μm) and incubated with DHE or DCFH-DA solution for 30 minutes at a temperature of 37°C. Sections were then rinsed with PBS and incubated with an anti-fluorescence quenching agent. The fluorescence intensities of cells and tissue sections were then visualized (excitation 590nm and emission 520 nm) using a fluorescence microscope (Zeiss AXIO Imager A1m). The fluorescence intensity of samples was quantified using Image J software (NIH, MD, USA). All tests were carried out three times.

### Evaluating Apoptosis by TUNEL Assay

Apoptosis in cardiac myocytes was evaluated using the TUNEL assay as the manufacturer’s protocol. After staining with the TUNEL reagents, the frozen rat heart sections were fixed, washed, and incubated with proteinase K for 30 minutes. Then, the sections were subjected to incubation with TdT/dUTP (1:9) enzyme reaction solution at a temperature of 37°C for 1 hour in darkness, and subsequently, exposed to DAB solution at room temperature for 10 min. Finally, images were abstained for each sample using the microscope (Olympus, Japan; magnification 400×).

### Statistical Analysis

All the results from the analyses are expressed as the mean ± standard error (SEM). All the statistical analyses were conducted utilizing the Graph Pad Prism 7.0 software (San Diego, CA, USA). The statistical significance of multiple comparisons was calculated by two-way ANOVA; the unpaired Student’s t-test was employed to compare two groups. *P* < 0.05 or *p* < 0.01 was considered to have significance.

## Results

### Effects of DOX on Rat Heart Tissues

**Figure 1:**
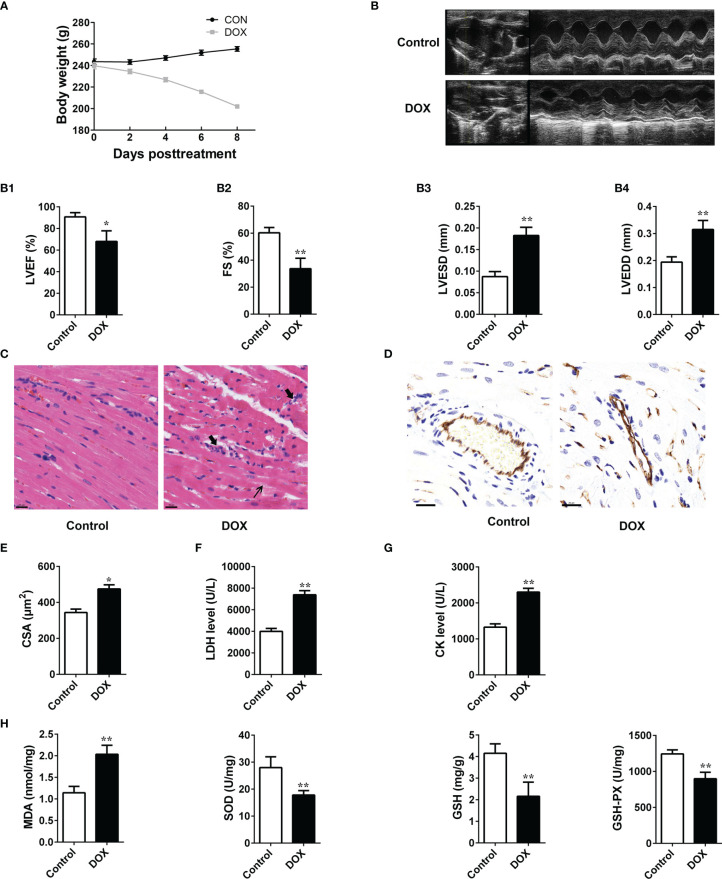
The impacts of DOX on the rat heart. **(A)** Daily body weight changes. **(B)** Representation of M-mode left ventricular (LV) echocardiogram. (B1) Left ventricular ejection fraction (LVEF), (B2) FS: fractional shortening, (B3) left ventricular end-systolic diameter (LVESD) and (B4), left ventricular end-diastolic diameter (LVEDD). **(C)** Representative images of HE-staining of rat heart tissues treated with or without doxorubicin (DOX) for 8 days. The structure of dysregulated myocytes is marked as thin arrowhead, inflammatory cell infiltration marked as thick arrowhead. Scale bars: 20 μm (center and right panels). **(D)** Representative photos of CD31 immunohistochemical staining in each group. Scale bars: 20 μm. **(E)** Cross-sectional areas (CSA) of cardiomyocytes in the LV. **(F, G)** Serum levels of LDH and CK in rats. **(H)** Determination of MDA, SOD, GSH and GSH-Px in the tissues. Data are presented as the mean ± SEM. *p < 0.05 and **p < 0.01 *vs* control.

During the experiment, we found that the rats in the DOX treated group showed inappetence, and less movement, and the DOX treatment reduced body weight gain in the animals (**Figure 1A**). tachypnea as compared to the control. Transthoracic echocardiography (**Figures 1B**) showed that cardiac dilatation was accompanied by cardiac dysfunction in DOX-treated rats. All the echocardiographic values of the DOX-treated rats reflected a decline in cardiac functions. LVEF and FS values decreased markedly, while those of LVESD and LVEDD increased remarkably after DOX treatment, as indicated by the M-mode echocardiograms (**Figure 1B**). Eight days after DOX injection, their hearts were excised, and H&E staining was performed. Histological examination suggested that the myocardial cells of the control rats were carefully organized and showed no abnormalities, while DOX-treated rats showed clusters of degenerating cardiomyocytes having extensive vacuolation and inflammatory infiltration (**Figure 1C**). As a marker of angiogenesis, CD31 was widely used in the research to investigate the microvessel (24) and was reduced significantly in the DOX treated group (**Figure 1D**). The cross-sectional areas (CSA) of cardiomyocytes in the LV increased after DOX treatment, which suggested the presence of cardiac hypertrophy in the DOX treated group (**Figure 1E**). Both CK and LDH levels increased significantly in the DOX group (**Figures 1F, G**). In addition, the levels of MDA in heart tissues increased remarkably in the DOX-treated rats, while those of GSH-Px, GSH, and SOD decreased significantly (**Figure 1H**). These results indicated that DOX could induce the HF in rats, and this process may be closely related to the oxidative damage.

### The Protein of PRR and RAC1 Was Upregulated in DOX-Treated Rats

**Figure 2:**
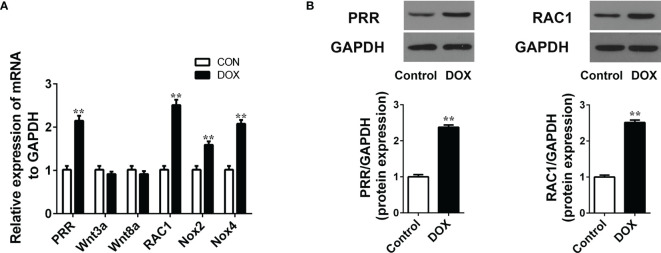
Doxorubicin treatment increases the mRNA/protein expression of PRR and RAC1. **(A)** RT-qPCR assay to evaluate the mRNA levels of PRR, Wnt3A, Wnt8a, RAC1, NOX2 and NOX4 in heart tissues (n = 3). **(B)** Western blotting and statistical analysis of the (pro)renin receptor (PRR) protein expression(n = 3). Data are presented as the mean ± SEM. **p < 0.01 *vs* control.

PRR and its related protein Wnt, RAC1 and NOX are involved in the cardiac dysfunction (11, 15). In this study, we detected the mRNA expression of these proteins in the DOX treated rats and found that DOX significantly affected the PRR mRNA levels, accompanied by significant upregulation in the expressions of RAC1, NOX2, and NOX4 (**Figure 2A**, *p* < 0.05), but Wnt3A and Wnt8A were not significantly affected (*p* > 0.05, **Figure 2A**). Furthermore, western blotting showed that the protein levels of PRR and RAC1 increased significantly by DOX treatment (**Figure 2B**), indicating that DOX induced HF was related to PRR and RAC1 expression.

### PRR Inhibition Reduces Oxidative Stress and Apoptosis in H9C2 Cells Treated With DOX

**Figure 3:**
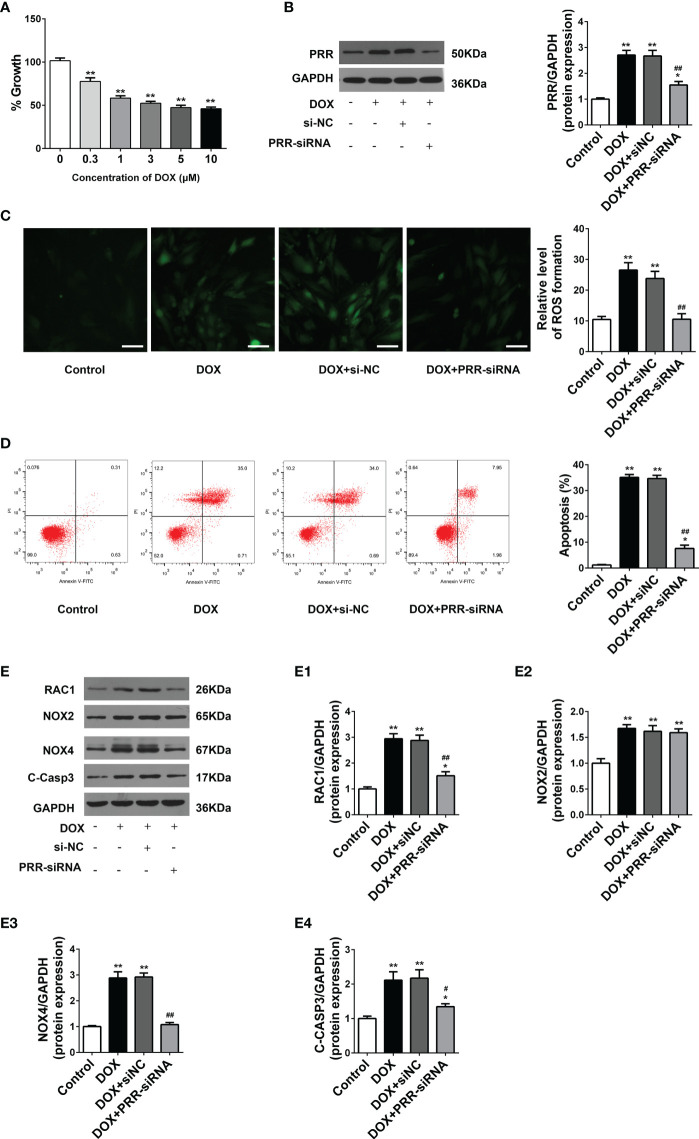
PRR inhibition downregulated the overexpression of oxidant species and apoptosis in H9C2 cells treated with doxorubicin. **(A)** Cell viability of H9C2 cells treated with Doxorubicin (DOX) treated at different concentrations (0–10 μM). **(B)** The protein expression of PRR downregulated by PRR-siRNA in H9C2 cells. (B1) Quantification of PRR/GAPDH protein expression levels relative to changing PRR expression. **(C)** Representative oxidative stress based on DHE relative fluorescence intensity of H9C2 cells. **(D)** Flow cytometry shows that PRR expression is directly related to cell apoptosis in doxorubicin (DOX)-treated H9C2 cells. **(E)** The protein expression of RAC1, NOX2, NOX4, and cleaved caspase3 was regulated by PRR inhibition in H9C2 cells. (E1)-(E4) Quantification of RAC1/GAPDH, NOX2/GAPDH, NOX4/GAPDH, and cleaved caspase3/GAPDH protein expression levels relative to changing PRR expression. (*p < 0.05, **p < 0.01 *vs* the control group. ^#^ *p* < 0.05, ^##^ *p* < 0.01*vs* DOX group).

H9C2 cells were employed to examine the impacts of PRR and RAC1 on DOX-induced myocardial cell injury. CCK-8 assay showed that cell viability reduced significantly in a dosage-dependent way following the DOX treatment (**Figure 3A**). Based on the results of optimization, we used 5 μM DOX to induce myocardial cell injury in the subsequent experiments. To further investigate the role of PRR-RAC1 pathway, the PRR-siRNA was used to silence the expression of the PRR (**Figure 3B**). Subsequently, ROS production and cell apoptosis were tested. As shown in **Figure 3C**, DOX treatment increased the ROS levels, and this impact was substantially attenuated by PRR-siRNA. In addition, flow cytometry suggested that cellular apoptosis decreased upon PRR-siRNA transfection in DOX-treated H9C2 cells (**Figure 3D**). These results demonstrated that PRR expression was significantly correlated with oxidative stress damage and cell apoptosis induced by DOX.

Next, we determined the expression of key proteins related to PRR in H9C2 cells to elucidate their underlying mechanisms. As shown in **Figure 3E**, DOX treatment significantly enhanced the expressions of RAC1, NOX4, NOX2, and cleaved caspase3 in H9C2 cells in contrast with the control (*p* < 0.05). These phenomena were reversed upon PRR silencing (**Figure 3E**). Moreover, as compared to the control group, PRR-siRNA down-regulated the expressions of RAC1 and NOX4 but not NOX2 in H9C2 cells (**Figure 3C**) (*p* < 0.05). Therefore, these data suggested that PRR could regulate RAC1 expression, thereby mediating DOX-induced myocardial oxidative stress injury.

### RAC1 Was the Downstream of PRR

RAC1 is required for the activation of the majority of the myocardial superoxide production complexes for their subsequent role in the activation of nicotinamide adenine dinucleotide phosphate oxidase (NADPH oxidase, NOX) (27). In cardiac hypertrophy (28, 29), hyperglycemia (30), and failing human myocardium (31), RAC1 plays an important role in regulating the myocardial superoxide production. Previous studies also suggest that the activation of RAC1 is involved in PRR-related myocardium injury (19). To further study the potential mechanism underlying the regulation of PRR on RAC1 pathway and the effect of RAC1 on DOX-induced myocardial cell injury, we silenced the RAC1 expression to evaluate the expression of relative proteins and oxidative stress levels in DOX-treated H9C2 cells.

**Figure 4:**
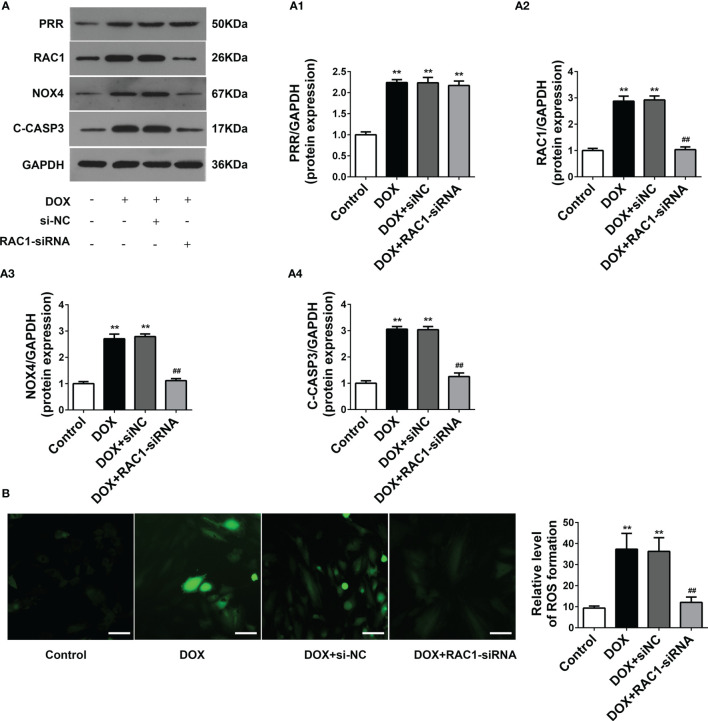
The effect of RAC1 inhibition on the regulation of ROS-related protein levels in doxorubicin (DOX)-treated H9C2 cells. **(A)** Protein expressions of PRR, RAC1, NOX4 and cleaved Caspase3 after RAC1 inhibition in H9C2 cells. (A1)-(A4) Quantification of PRR/GAPDH, RAC1/GAPDH, and NOX4/GAPDH levels with a change in RAC1 expression. **(B)** Representative oxidative stress levels as determined by the DCFH-DA assay. (**p < 0.01 *vs* the control group. ^##^ *p* < 0.01 *vs* DOX group).

RAC1-siRNA knockdown was used to silence the expression of RAC1 in H9C2cells. RAC1-siRNA may substantially reduce the protein expression level of RAC1(**Figure 4A**). As opposed to the DOX treatment group, NOX4 expressions were also downregulated after RAC1-siRNA knockdown, however, the protein level expression of PRR in H9C2 cells was not affected after RAC1-siRNA transfection (**Figure 4A**). Combined the above results (**Figure 3**), we reasonably concluded that PRR was upstream of RAC1. As shown in **Figure 4B**, ROS production increased after DOX treatment, which was subsequently reversed by RAC1-siRNA transfection.

### Pharmacological Inhibition of PRR/RAC1 Ameliorates DOX-Induced Cardiac Hypertrophy and Cardiac Dysfunction

**Figure 5:**
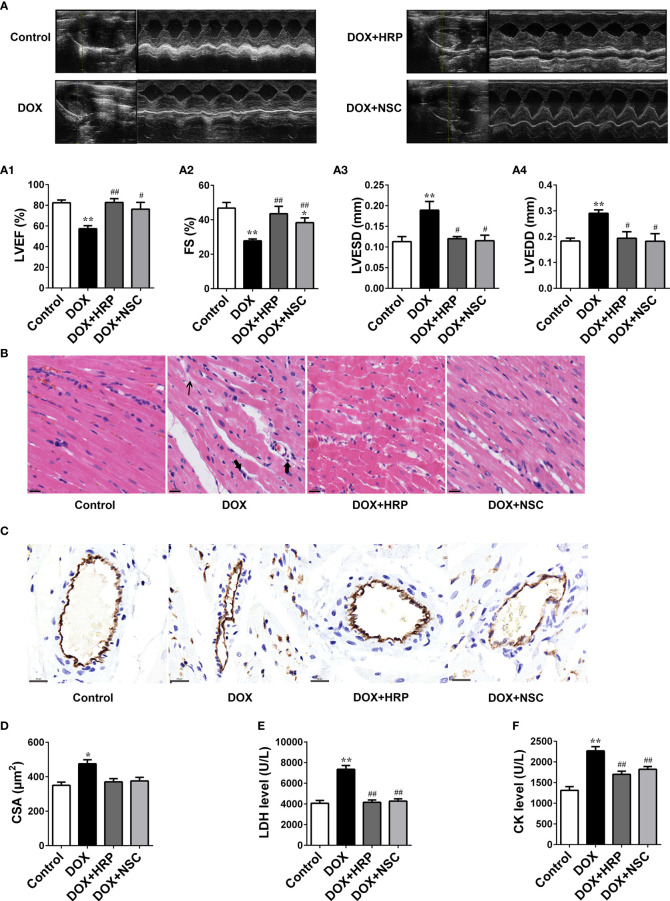
Therapeutic effect of PRR/RAC1 inhibition on doxorubicin-induced HF in rats. Protective effects of pharmacological (pro)renin receptor (PRR) inhibition on doxorubicin (DOX)-induced cardiac hypertrophy and dysfunction. **(A)** Representative M-mode left ventricle (LV) echocardiogram. (A1) Left ventricular ejection fraction (LVEF). (A2) Fractional shortening (FS); (A3) left ventricular end-systolic diameter (LVESD). (A4) left ventricular end-diastolic diameter (LVEDD). **(B)** Representative photomicrographs of hematoxylin-eosin stained left ventricle (LV) sections. The structure of dysregulated myocytes is marked as thin arrowhead, inflammatory cell infiltration marked as thick arrowhead. **(C)** Representative photos of CD31 immunohistochemical staining in each group. Scale bars: 20 μm. **(D)** Cross-sectional areas (CSA) of cardiomyocytes in the LV. **(E, F)** Serum levels of LDH and CK in rats. Data are presented as the mean ± SEM. *p < 0.05 and **p < 0.01 *vs* control; ^#^ *p* < 0.05, ^##^ *p* < 0.01 *vs* the DOX group.

To further verify the effect PRR/RAC1 in DOX-induced HF, rats were treated using the PRR inhibitor HRP, and the RAC1 inhibitor NSC23766, after the DOX challenge. Cardiac dysfunction induced by DOX was ameliorated significantly upon treatment with HRP and NSC23766 (**Figure 5**). Next, transthoracic echocardiography showed that inhibition of PRR or RAC1 reversed the abnormal levels of LVEF, FS, LVESD, and LVEDD after DOX treatment in rats, thereby suggesting that inhibition of PRR or RAC1 further ameliorated cardiac dysfunction challenged by DOX in rats (**Figure 5A**). As illustrated in **Figure 5B**, DOX treatment caused substantial inflammatory, cell infiltration, and pyknosis, which was improved significantly upon HRP or NSC23766 treatment. To further investigate the effect of PRR/RAC1 inhibition on vascular structures, the immunohistochemistry of CD31 was detected. As shown in **Figures 5C**, staining for CD31 showed a significant increase in the number of capillaries after PRR or RAC1 inhibition in comparison to DOX treated rats. Moreover, CSA of cardiomyocytes in the LV region decreased by DOX treatment and could be partly reversed by treatment with HRP or NSC23766 (**Figure 5D**). Both the increased CK and LDH levels were downregulated by PRR or RAC1 inhibition (**Figures 5E, F**). Taken together, these findings suggested that abnormal expression of PRR/RAC1 was related to the pathogenesis of cardiac hypertrophy and cardiac dysfunction in DOX induced HF.

### Pharmacological Inhibition of PRR/RAC1 Ameliorates ROS Production and Apoptosis in Rats With DOX-Induced HF

**Figure 6:**
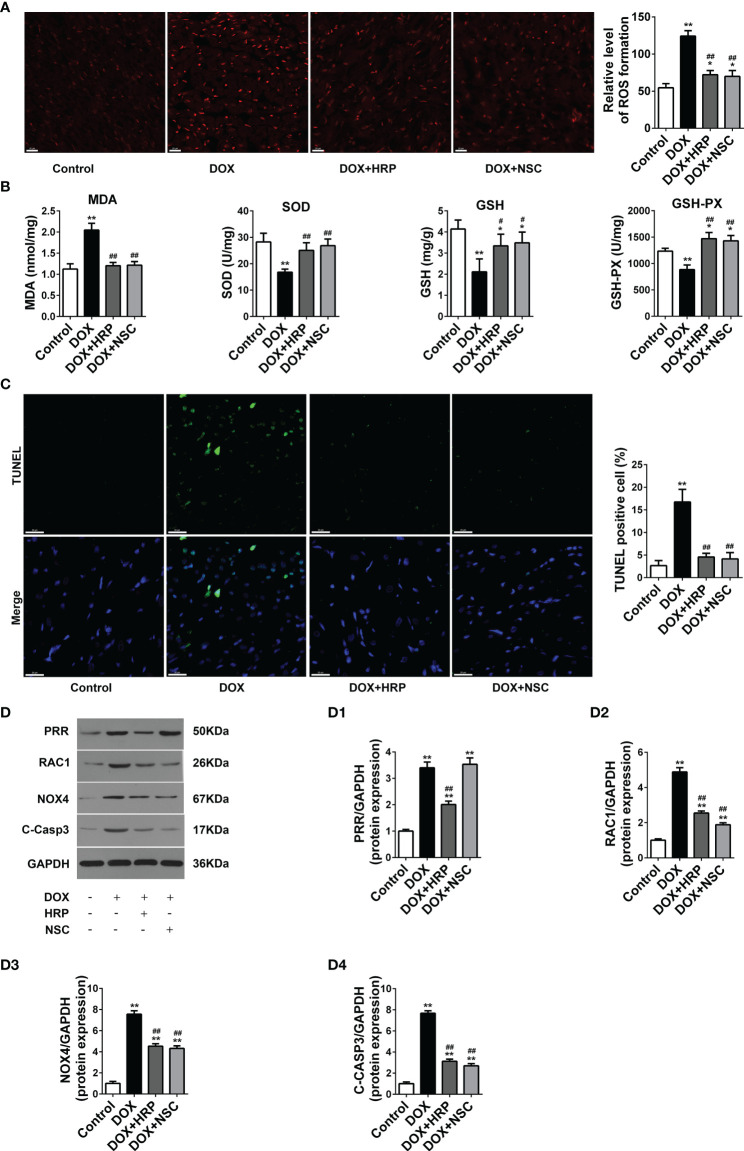
Effects of handle region peptide (HRP) and RAC1 inhibitor, NSC23766 (NSC) on ROS production and cardiotoxicity in doxorubicin (DOX)-induced HF rats. **(A)** Representative photomicrographs of DHE stained of the LV. **(B)** Determination of MDA, SOD, GSH, and GSH-PX in heart tissues. **(C)** Representative photomicrographs of TUNEL stained LV sections, where blue fluorescence denotes the position of the nucleus and green fluorescence denotes apoptotic cells; Scale bars, 50 μm. **(D)** The protein expressions of PRR, RAC1, NOX4 and cleaved caspase3 in the hearts of doxorubicin (DOX)-treated rats. (D1)-(D4) Statistical analysis of the protein expressions of PRR, RAC1, NOX4 and cleaved caspase3 (C-Casp3); GAPDH was used as the loading control. The bar graph shows the results of densitometric analysis (n = 3 per group). Data are presented as the mean ± SEM, *p < 0.05 and **p < 0.01 *vs* control; ^#^ *p* < 0.05 vs the DOX group, ^##^ *p* < 0.01 *vs* the DOX group.

Next, we assessed the impact of PRR/RAC1 inhibition on oxidative stress levels and apoptosis in DOX-induced HF rats. In addition to alleviating the pathology reconstruction, HRP or NSC237666 could reduce the production of superoxide radicals induced by DOX (**Figures 6A, B**). Thus, these results indicated that PRR or RAC1 inhibition could significantly alleviate DOX-induced oxidative damage *in vivo*. To reliably evaluate cardiac cell damage and recovery in DOX-related HF, TUNEL staining was used to test the cytotoxicity and visually evaluate the effects of HRP and NSC23766. As illustrated in **Figure 6C**, the proportion of TUNEL positive cells increased markedly after DOX treatment, thereby indicating the occurrence of cell apoptosis of myocardial cytotoxicity. In contrast, both HRP and NSC23766 could alleviate cardiomyocytes from apoptosis to some extent. Taken together, these results indicated that inhibition of PRR or RAC1 could significantly protect the heart tissues from HF. Western blotting also showed that treatment with HRP or NSC23766 inhibited the DOX-related activation of PRR or RAC1, respectively (**Figure 6D**). Moreover, PRR remained unaffected in NSC23766-treated rats. In addition, the increased expression of cleaved caspase3 was reversed upon inhibition of PRR or RAC1.

## Discussion

Since the late 1960s, DOX has been widely used in acute leukemia, breast cancer, lymphoma, ovarian cancer treatment (32). Unfortunately, the cardiotoxicity of DOX can lead to structural and functional changes of heart and even HF (26, 33). Several studies suggest that the underlying mechanism of DOX-induced heart injury is correlated with the level of oxidative stress (34–36), however, the potential specific mechanisms underlying DOX-triggered oxidative injury remain unknown.

PRR is a receptor that consists of 350 amino acids residues and performs multiple functions (11). Recent studies showed that PRR plays an important role in a variety of myocardial diseases. In an earlier research report, PRR was suggested to be related to myocardial remodeling after *in situ* injection of the PRR gene into the heart (37). In the hearts of rats with diabetes, both PRR mRNA and protein level expressions showed a marked increase, thereby aggravating myocyte hypertrophy and deterioration of cardiac function (38). In post-myocardial infarction heart, PRR exacerbates myocardial fibrosis and deteriorates the cardiac function independent of Ang II (39). But the role of PRR in the DOX induced HF is still unknown. In the current study, we found that DOX stimulated severe myocardial dysfunction and oxidative injury along with PRR overexpression in rats. Our *in vivo* experiments also suggested that silencing of PRR could alleviate DOX-related endothelial injury and then heart injury *via* reducing oxidative stress (**Figure 5**). Therefore, these evidence suggested that PRR played a critical role in DOX-induced HF.

PRR can function as an accessory subunit linking the vacuolar proton pump, V-ATPase, and the low-density lipoprotein receptor-related protein 6 (LRP6), a coreceptor of Wnt receptor (40, 41). Recent studies found that PRR is involved in the transduction of various signals through the classic Wnt signaling pathway and exacerbates kidney damage by amplifying the Wnt signaling cascade (40, 41). However, the mRNA expressions of Wnt3A and Wnt8A did not increase in the DOX-induced HF in our study. Therefore, PRR may play roles *via* other mechanisms underlying DOX-induced HF.

As a critical functional constituent of the local tissue RAS, the pathophysiological roles of PRR have been widely studied. Previous study showed that prorenin induced cytoskeleton reorganization by activating the intracellular RAC1 (19). RAC1 inhibition is found to be relevant to the cardioprotective effects of lovastatin in mice (42). Our research also showed that the RAC1 expression was increased accompanied with PRR overexpression. Both *in vitro* and *in vivo* findings showed that the RAC1 inhibition was effective in alleviating oxidative stress in cardiac cells, thereby resulting in an attenuation of cardiac hypertrophy and dysfunction. Moreover, the inhibition of PRR downregulated the expression of RAC1, however, the inhibition of RAC1 did not significantly affect the expression of PRR (**Figures 4**, **6**), thereby suggesting a pathological role of PRR-mediated RAC1 activation in DOX-induced HF.

By binding to guanosine triphosphate (GTP) and migrating to the membrane with a core cytosolic complex, RAC1 is required for the activation of NOX, the leading source of superoxide production complex in the myocardium (27, 43). NOX–derived ROS plays a crucial role in several cardiovascular diseases (11, 44–46). With respect to all the isoforms of NOX, NOX4 and NOX2 are the major isoforms in the myocardium and play different roles in cardiac injury: NOX2 is primarily localized on the plasma membrane, whereas NOX4 is found primarily on intracellular membranes, mitochondria, the endoplasmic reticulum or the nucleus (47–49). A previous study indicates that NOX4 is the leading source of ROS production in a failing heart (50). In our study, both NOX2 and NOX4 expressions were upregulated in DOX-treated rats, thereby suggesting their role in the pathological processes. These findings were consistent with the previously published results (32). Whereas the suppression of PRR downregulated NOX4 but not NOX2 expression (**Figure 3C**), we thus reasonably suggest the presence of another pathway apart from PRR-RAC1 in NOX2 regulation which needs further study. In a study by PAN L L et al., knockdown of NOX4 was shown to attenuate myocardial fibrotic responses and intercellular ROS generation in cardiac fibroblasts (51). We found PRR or RAC1 inhibition reduced NOX4 expression both *in vitro* and *in vivo*, which suggested a critical function of NOX4 in the PRR-RAC1 pathway in DOX-related HF. These data suggested that the elevated PRR in DOX-induced HF rats might activate NOX4 *via* the RAC1 pathway, and subsequently aggregate oxidative injury.

## Conclusion

In conclusion, we found that PRR plays an important role in DOX-induced HF, and cardiac function significantly improved upon the inhibition of PRR or its downstream RAC1, as indicated by the improvement in the levels of oxidative stress and histopathological changes. Thus, our findings suggested a candidate treatment approach for attenuating oxidative stress, thereby mitigating myocardial damage during DOX treatment.

## Data Availability Statement

The original contributions presented in the study are included in the article. Further inquiries can be directed to the corresponding authors.

## Ethics Statement

The animal study was reviewed and approved by Animal Ethics Committee of Tongji Medical College, Huazhong University of Science and Technology (Wuhan, China).

## Author Contributions

X-yD and D-cX designated the study, performed the experiments, analyzed the data and wrote the manuscript. PG contributed to data collection and partly analyzed the data too. Y-lL and HP supervised the study. All authors contributed to the article and approved the submitted version.

## Conflict of Interest

The authors declare that the research was conducted in the absence of any commercial or financial relationships that could be construed as a potential conflict of interest.

## Publisher’s Note

All claims expressed in this article are solely those of the authors and do not necessarily represent those of their affiliated organizations, or those of the publisher, the editors and the reviewers. Any product that may be evaluated in this article, or claim that may be made by its manufacturer, is not guaranteed or endorsed by the publisher.
